# Field‐evolved resistance to imidacloprid and ethiprole in populations of brown planthopper Nilaparvata lugens collected from across South and East Asia

**DOI:** 10.1002/ps.3980

**Published:** 2015-02-19

**Authors:** William T Garrood, Christoph T Zimmer, Kevin J Gorman, Ralf Nauen, Chris Bass, Thomas GE Davies

**Affiliations:** ^1^ Biological Chemistry and Crop Protection DepartmentRothamsted ResearchHarpendenHertfordshireUK; ^2^ Oxitec LtdOxfordUK; ^3^ Bayer CropScience AGPest Control BiologyMonheimGermany

**Keywords:** brown planthopper, resistance, cytochrome P450, insecticides

## Abstract

**BACKGROUND:**

We report on the status of imidacloprid and ethiprole resistance in Nilaparvata lugens **Stål** collected from across South and East Asia over the period 2005–2012.

**RESULTS:**

A resistance survey found that field populations had developed up to 220‐fold resistance to imidacloprid and 223‐fold resistance to ethiprole, and that many of the strains collected showed high levels of resistance to both insecticides. We also found that the cytochrome P450 CYP6ER1 was significantly overexpressed in 12 imidacloprid‐resistant populations tested when compared with a laboratory susceptible strain, with fold changes ranging from ten‐ to 90‐fold. In contrast, another cytochrome P450 CYP6AY1, also implicated in imidacloprid resistance, was underexpressed in ten of the populations and only significantly overexpressed (3.5‐fold) in a single population from India compared with the same susceptible strain. Further selection of two of the imidacloprid‐resistant field strains correlated with an approximate threefold increase in expression of CYP6ER1.

**CONCLUSIONS:**

We conclude that overexpression of CYP6ER1 is associated with field‐evolved resistance to imidacloprid in brown planthopper populations in five countries in South and East Asia. © 2015 The Authors. Pest Management Science published by John Wiley & Sons Ltd on behalf of Society of Chemical Industry.

## 1 INTRODUCTION

The brown planthopper (BPH), *Nilaparvata lugens* Stål, is an economically important pest of rice throughout both tropical and temperate zones of South and East Asia. It causes damage to the rice crop via direct phloem‐sap feeding, leading to nutrient depletion within the plant, which when infestation levels become high enough manifests as a characteristic stunting, wilting and browning of the affected crop, often referred to as ‘hopperburn’. BPH is also an effective vector of a number of rice pathogens, including ragged stunt virus and grassy stunt virus.^1^ The resulting cumulative damage to the rice crop can result in a significant (up to 60%) loss of yield in susceptible rice varieties.^2^ This is starkly illustrated by the observation that, between 2009 and 2011, rice production in Thailand suffered huge losses due to BPH, with more than 3 million ha infested and in excess of 1.1 million t of paddy, with an export value of an estimated $US 275 million, lost (data published by the International Rice Research Institute).

The control of BPH has for many years predominantly relied on the use of synthetic insecticides. This has resulted in the emergence of populations with high levels of resistance to many of the major classes of insecticides, including the organophosphates, carbamates, pyrethroids, neonicotinoids and phenylpyrazoles.^3^, ^4^, ^5^, ^6^ Since the early 1990s, the neonicotinoid insecticide imidacloprid has been widely applied throughout Asia for BPH control. Reduced efficacy/resistance to this insecticide emerged in populations across Asia over the period 2003–2006.^7^, ^8^ More recent monitoring across nine regions of China showed that imidacloprid resistance levels have again increased, with resistance ratios [LD_50_ field population/LD_50_ susceptible (1995 collected) strain] as high as 617‐fold being recorded in 2012.^6^ Similar levels of imidacloprid resistance in BPH immigrating into Japan have recently been reported, with resistance ratios of 616‐fold (comparing LD_50_ values of populations sampled in 1992 to 2012).^9^ Owing to the significant resistance to neonicotinoid insecticides, phenylpyrazole (fiprole) insecticides, such as ethiprole and fipronil, which target the gamma‐aminobutyric acid (GABA)‐gated chloride channel of the insect's central nervous system,^10^ have increasingly been used as a substitute for BPH control. However, emerging resistance to fipronil (23.8–43.3‐fold resistance) and cross‐resistance (47.1–100.9‐fold) to ethiprole in field populations of BPH have been reported in China,^11^, ^12^ and significant (308.5‐fold) levels of resistance to ethiprole in Thailand.^5^

Although the molecular mechanism(s) underlying resistance to fiproles have not been fully characterised,^5^ significant progress has been made in characterising the molecular basis of resistance to imidacloprid. Target‐site resistance to this compound was described in a laboratory‐selected strain of BPH before reports of control failure in the field; however, this mechanism has never been identified in any field‐collected population.^13^ In contrast, several studies have provided evidence that enhanced cytochrome P450 monooxygenase (P450) activity contributes to the neonicotinoid resistance of field‐collected populations of BPH.^14^, ^15^, ^16^ This detoxification mechanism was initially implicated by use of the metabolic enzyme inhibitor piperonyl butoxide (PBO) and the model substrate 7‐ethoxycoumarin.^15^, ^17^ More recently the overexpression of two candidate P450 enzymes, CYP6ER1 and CYP6AY1, has been linked with imidacloprid resistance.^18^, ^19^ In the first study, the expression levels of 32 tentative unique P450s, identified from two recent sequencing projects and by degenerate PCR, were examined in a susceptible *N. lugens* strain and moderately and highly resistant strains from China and Thailand, using quantitative real‐time PCR. A single P450 gene, CYP6ER1, was identified as highly overexpressed (up to 40‐fold) in all resistant strains compared with the susceptible strain, and the level of expression observed in the different strains was significantly correlated with the resistance phenotype.^18^ In the second study, the expression levels of 14 P450 genes were compared between a laboratory strain selected with imidacloprid for 40 generations and a susceptible strain, using quantitative RT‐PCR. Six genes were identified as significantly overexpressed in the resistant strain, with CYP6AY1 showing the highest level of overexpression (∼18‐fold) compared with the susceptible strain.^19^ Functional expression of CYP6AY1 and RNAi experiments provided evidence that CYP6AY1 has the capacity to metabolise imidacloprid and confer resistance.^19^

The aim of the present study was to analyse the changing levels of resistance to imidacloprid and ethiprole in *N. lugens* field strains collected from five countries in South and East Asia from 2005 through to 2012, and to investigate the relative roles of CYP6ER1 and CYP6AY1 in the resistance of these strains to imidacloprid.

## 2 EXPERIMENTAL METHODS

### 2.1 Insect strains

Baseline susceptibility data were generated using a laboratory‐maintained strain of *N. lugens* (Bayer‐S) provided by Bayer CropScience (Monheim, Germany). Bayer CropScience also organised the transfer to Rothamsted Research of field strains collected from across South and East Asia between 2005 and 2012. All strains were reared in the laboratory on whole rice plants (*Oryza sativa* L. ssp.) under controlled environmental conditions (26 °C/16 h photoperiod).

### 2.2 Laboratory selection

Two of the field strains, NL9 and NL39, demonstrating relatively high levels of resistance to imidacloprid, were placed under further selection with imidacloprid in the laboratory. NL9 was reared on rice plants treated with successively higher doses (concentrations ranging between 10 and 180 mg L^−1^) of imidacloprid over 13 generations, whereas NL39 was placed directly onto rice plants treated with 200 mg L^−1^ imidacloprid and selected over two generations.

### 2.3 Topical application bioassay (imidacloprid)

Adult macropterous (long‐winged) females of *N. lugens* were taken from age‐structured populations and were less than 10 days old. Approximately 15 females were lightly anaesthetised and dosed with the required concentration of technical imidacloprid on the upper surface (pronotum) of the prothorax using 0.25 µL of acetone as the solvent carrier, delivered using a hand‐held Burkard microapplicator (Burkard Manufacturing Co. Ltd, Rickmansworth, UK) fitted with a 1 cm^3^ all‐glass syringe. Control insects were dosed with 0.25 µL of acetone only. Treated individuals were placed in 50 mL specimen tubes containing untreated five‐week‐old rice stems (cut into 10 cm lengths) and contained using a ventilated lid. A small hole (3 mm diameter) was drilled in the base of each of the tubes, which were then stored vertically in a water bath (submerging only the base of each rice stem) in a 16 h photoperiod at 26 °C for 48 h. Insect mortality at 48 h was assessed by eye; adults showing no sign of movement were scored as dead. Bioassays consisted of three replicates at each concentration. Diagnostic doses represented the LD_95_ (4 mg L^−1^) and 5 × LD_95_ (20 mg L^−1^) of the susceptible strain.

### 2.4 Leaf‐dip bioassay (ethiprole)

Adult females were taken from age‐structured populations and were less than 10 days old. Rice stems (10 cm cut lengths) were dipped into the required concentrations of formulated fiprol insecticide for 20 s, air dried and placed in a plastic specimen tube. Approximately 15 females were aspirated directly into each of the tubes, which were sealed with a ventilated lid. A small hole (3 mm diameter) was drilled in the base of each of the tubes, which were then stored vertically in a water bath (submerging only the base of each stem) in a 16 h photoperiod at 26 °C for 72 h. Mortality was assessed by eye; adults showing no sign of movement were scored as dead. Bioassays consisted of three replicates at each concentration.

### 2.5 Data analysis

Probit analysis with Genstat 16th Edition software (VSN International Ltd, Hemel Hempstead, UK) was conducted to generate estimated LC_50_ values. Resistance factors were calculated by dividing the LC_50_ of a resistant strain by that of the susceptible strain. Mortality rates at diagnostic concentrations were subjected to Abbott's correction for natural mortality.^20^ Standard errors for mortalities at diagnostic concentrations were calculated using a binomial model.

### 2.6 Real‐time quantitative RT PCR

In qRT‐PCR analysis of CYP6ER1 and CYP6AY1 expression, primers designed previously^18^ and the CYP6AY1 primers employed by Ding *et al.* 19 were used. PCR reactions (15 µL) contained 5 µL of cDNA (2.5 ng), 7.5 µL of SYBR Green JumpStart Taq Readymix (Sigma Aldrich) and 0.25 µM of each primer. Samples were run on a Rotor‐Gene 6000 (Corbett Research, Cambridge, UK) using the following temperature cycling conditions: 10 min at 95 °C, followed by 40 cycles of 95 °C for 15 s, 57 °C for 15 s and 72 °C for 20 s. A final melt‐curve step was included post‐PCR (ramping from 72 to 95 °C by 1 °C every 5 s) to check for non‐specific amplification. Each qRT‐PCR experiment consisted of three independent biological replicates, with two technical replicates for each. Technical replication was limited to two replicates, (1) as PCR reactions were set up using a liquid handling robot (CAS 1200; Corbett Research) which provided high levels of technical reproducibility, and (2) to allow us to employ a sample maximisation strategy (i.e. running as many samples as possible in the same run in order to minimise technical run‐to‐run variation). Data were analysed according to the ΔΔ*C*_t_ method.^21^ For normalisation, two reference genes were validated experimentally for each strain, actin and *α*2‐tubulin, with the geometric mean of the selected genes then used for normalisation according to the strategy described previously.^22^

## 3 RESULTS AND DISCUSSION

### 3.1 Development of imidacloprid resistance in N. lugens populations from 2005 to 2012

As previously reported,^7^ responses of 2005 field‐collected samples of *N. lugens* to imidacloprid showed variation, particularly at the lower (4 mg L^−1^) dose tested (Table 1), with some strains appearing susceptible but other strains showing the first indications of a resistance problem. Strains collected that exhibited a decreased susceptibility to imidacloprid at the higher (20 mg L^−1^) diagnostic concentration (IND‐6 and IND‐7) were analysed for the presence of the Y151S mutation, known to reduce the agonist potency of a range of neonicotinoid insecticides, including imidacloprid.^13^ Using PCR‐based techniques, it was shown that, at the Y151S mutation site, individuals of both strains expressed ‘wild‐type’ base pairings, i.e. there was no evidence for Y151S‐mediated target‐site resistance as recently described for a laboratory‐selected strain.

**Table 1:** Mortalities (%) (± standard error) for all Nilaparvata lugens strains at two diagnostic doses (LD_95_ and 5 × LD_95_ of the susceptible strain) of imidacloprid topically applied to adult females. Highlighted data were previously reported in Gorman et al. 7

Strain	Year	Country of origin	Region/area	Imidacloprid^a^	
				4 mg L^−1^, 1 ng AI insect^−1^	20 mg L^−1^, 5 ng AI insect^−1^
Bayer‐S	—	—		91.43(±4.48)	100.00 ± nc
CHN‐1	2005	China	Nanjing	53.45(±6.39)	100.00 ± nc
IND‐1	2005	India	East Godavari District, Andhra Pradesh	85.21(±4.74)	100.00 ± nc
IND‐2	2005	India	Karnataka State	91.23(±3.68)	100.00 ± nc
IND‐3	2005	India	Mumbai	59.32(±6.09)	100.00 ± nc
IND‐4	2005	India	West Godavari District, Andhra Pradesh	83.34(±5.02)	100.00 ± nc
IND‐5	2005	India	Bellary District, Karnataka State	59.66(±7.57)	100.00 ± nc
IND‐6	2005	India	West Godavari District, Andhra Pradesh	17.98(±4.66)	81.50(±4.74)
IND‐7	2005	India	East Godavari District, Andhra Pradesh	18.63(±4.79)	71.40(±5.61)
ISA‐1	2005	Indonesia		96.36(±2.50)	100.00 ± nc
MAL‐1	2005	Malaysia		54.03(±6.91)	nt
THAI‐1	2005	Thailand		87.01(±4.20)	100.00 ± nc
VTN‐1	2005	Vietnam		92.10(±3.67)	100.00 ± nc
CHN‐2	October 2006	China	Guandong Province	41.41(±7.11)	46.20(±10.40)
CHN‐3	October 2006	China	Guangxi Province	23.34(±6.24)	75.81(±6.69)
CHN‐4	September 2006	China	Jiangsu Province	55.71(±6.64)	75.11(±6.92)
CHN‐5	October 2006	China	Hunan Province	35.00(±6.88)	67.50(±6.76)
IND‐8	April 2006	India	Bellary District, Karnataka State	57.53(±8.13)	97.14(±2.36)
IND‐9	April 2006	India	East Kolkata, West Bengal	50.00(±7.45)	79.71(±5.15)
IND‐10	October 2006	India	West Godavari, Andhra Pradesh	33.67(±6.68)	48.04(±5.97)
IND‐11	October 2006	India	East Godavari, Andhra Pradesh	0.00 ± nc	5.75(±4.18)
MAL‐2	December 2006	Malaysia	Sabak Bernam District, Selangor	13.87(±6.78)	33.07(±5.23)
THAI‐2	August 2006	Thailand	Chainat Province, San Buri District	22.41(±7.74)	35.71(±8.10)
THAI‐3	August 2006	Thailand	Suphanburi Province	35.00(±6.88)	67.50(±6.76)
VTN‐2	August 2006	Vietnam	Đ 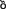 ng Tháp Province, Tháp M 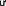 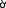 i District	2.27(±2.52)	0.00 ± nc
VTN‐3	August 2006	Vietnam	Long An Province, B 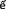 n L 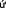 c District	26.63(±7.70)	42.11(±7.81)
NL2	October 2008	India	Bellary District, Karnataka State	100.00 ± nc	83.33(±6.80)
NL3	October 2008	India	Karnataka State	66.67(±8.61)	75.00(±7.91)
NL5	October 2008	Thailand	Samchuk District, Suphanburi Province	86.67(±6.21)	93.33(±4.55)
NL6	October 2008	India	West Medinapuri, West Bengal, East India	51.11(±8.33)	91.75(±4.35)
NL8	December 2008	Vietnam	Tantru District, Long An Province	64.29(±8.75)	82.14(±6.99)
NL9	August 2009	Thailand		26.92(±8.10)	53.85(±9.10)
NL10	September 2009	Indonesia	Subang, West Java	11.90(±5.40)	73.57(±7.45)
NL11	October 2009	India	Sindhanoor, Southern India	6.67(±4.55)	23.33(±7.72)
NL12	October 2009	India	Karnataka State	0.00 ± nc	7.69(±4.87)
NL13	October 2009	India	Nadia District, West Bengal, East India	0.00 ± nc	3.70(±3.45)
NL14	October 2009	India	Hooghly District, West Bengal, East India	0.00 ± nc	68.00(±8.52)
NL15	September 2009	China	Nanning City, Guangxi Province	34.38(±8.67)	82.36(±6.85)
NL16	September 2009	China	Danyang City, Jiangsu Province	2.32 ± nc	3.43 ± nc
NL17	November 2009	China	Wuhan City, Hubei Province	24.24(±7.14)	91.98(±4.66)
NL18	November 2009	China	Fengxin County, Jiangxi Province	39.50(±6.85)	86.15(±6.01)
NL19	December 2009	Indonesia	East Java	0.00 ± nc	25.93(±8.00)
NL20	December 2009	Indonesia	Gabus Pati District, Central Java	10.71(±5.65)	60.71(±8.92)
NL21	March 2010	Thailand	Suphanburi Province, Sriprachan District	16.78(±6.41)	7.05(±3.69)
NL25	October 2010	India	Koppal District, Karnataka State	16.27(±5.26)	37.00(±5.04)
NL27	September 2010	China	Danyang City, Jiangsu Province	66.52(±6.04)	83.26(±4.78)
NL28	September 2010	China	Nanning City, Guanxi Province	68.07(±6.95)	76.48(±6.19)
NL29	October 2010	India	West Bengal	85.51(±5.25)	85.03(±5.14)
NL30	September 2010	China	Nanchang City, Jianxi Province	62.40(±7.96)	79.35(±5.78)
NL31	October 2010	Taiwan	Yulin County	9.94(±4.85)	37.32(±7.75)
NL32	October 2010	China	Foshan City, Guandong Province	75.18(±6.30)	74.64(±6.42)
NL33	November 2010	Vietnam	Trà Vinh Province, Southern Vietnam	2.86(±2.64)	21.80(±6.70)
NL34	April 2011	India	Koppal District, Karnataka State	61.38(±6.34)	85.96(±4.52)
NL35	April 2011	India	Koppal District, Karnataka State	100.00 ± nc	96.50(±2.56)
NL39	August 2011	Vietnam	Hau Giang	2.00 ± nc	1.00 ± nc
NL40	August 2011	Indonesia	Anjatan District, Indramayu	8.90(±4.96)	27.32(±7.53)
NL41	August 2011	Indonesia	Binong District, Subang	23.75(±6.42)	45.67(±8.08)
NL42	August 2011	Indonesia	Gegesik District, Cirebon	26.67(±6.99)	41.09(±7.50)
NL43	August 2011	Indonesia	Binong District, Subang	19.44(±5.90)	46.02(±7.51)
NL44	August 2011	Indonesia	Parnanukan District, Subang	2.48(±2.52)	4.45(±3.22)
NL45	September 2011	India	Raipur, Chhattisgarth	30.14(±8.11)	34.25(±8.14)
NL46	October 2011	India	Mohanpur, West Bengal	10.00(±5.30)	28.00(±8.20)
NL47	September 2011	China	Xi Jiao District, Danyang City, Jiangsu Province	18.92(±6.44)	50.00(±8.45)
NL52	March 2012	India	Koppal District, Karnataka State	15.13(±5.67)	58.31(±8.00)
NL53	March 2012	India	West Godavari District, Andhra Pradesh	45.88(±7.88)	67.00(±7.34)
NL54	March 2012	India	Karimnagar, Warangar District	36.83(±7.58)	78.06(±6.63)
NL55	April 2012	India	East Godavari District, Andhra Pradesh	40.00(±7.95)	60.00(±7.95)
NL56	April 2012	India	East Godavari District, Andhra pradesh	62.11(±7.77)	67.33(±7.24)
NL57	October 2012	India	Kanagala District, Karnataka State	24.51(±7.38)	31.06(±6.98)
NL58	October 2012	India	Mudhapur, Karnataka State	29.41(±7.03)	32.86(±6.93)
NL59	October 2012	India	Sidhikerra, Karnataka State	15.74(±5.69)	42.42(±8.02)

a nt = not tested; nc = not calculable.

In contrast to 2005, all 13 field samples collected in 2006 showed reduced susceptibility to imidacloprid at both diagnostic doses. Responses at 4 mg L^−1^ ranged from 0 to 60% mortality, and those at 20 mg L^−1^ from 0 to 97% mortality. The most resistant samples, IND‐11, VTN‐2, MAL‐2, THAI‐2 and CHN‐2, originated from different countries (India, Vietnam, Malaysia, Thailand and China), leading to the conclusion that resistance was neither confined to nor focused within a specific geographical region. This widespread distribution is, however, consistent with the migratory behaviour of *N. lugens*. To assess the potency of the mechanism(s) responsible, dose–response data for one of the most resistant samples (IND‐11) were generated. A comparison between the laboratory susceptible strain (S) and strain IND‐11 showed near‐parallel response lines,^7^ with a resistance ratio of 96.7 at LD_50_. Approximately 30% of the IND‐11 individuals were capable of surviving 100 mg L^−1^ (25 ng AI insect^−1^), which relates to 25 × LD_95_ of the susceptible strain. As in 2005, results for two of the most imidacloprid‐resistant strains collected in 2006 (CHN‐2 and THAI‐2) also disclosed ‘wild‐type’ sequences at the Y151S mutation site.

A limited number of field samples collected in 2008 from India, Thailand and Vietnam suggested that resistance was not as high in individual strains as in 2006. However, for field samples collected in 2009, responses at 4 mg L^−1^ ranged from 0 to 40% mortality, and those at 20 mg L^−1^ from 4 to 92% mortality. Again, highly resistant samples were identified as originating from different countries (India, China, Indonesia and Thailand), suggesting that the resistance problem across South and East Asia had not really abated. LD_50_ analysis of strain NL9 from Thailand indicated a resistance ratio of 139 (Table 2), roughly comparable with that reported for IND‐11 in 2006. Resistance to imidacloprid appeared to stabilise in 2010, but a highly resistant sample NL30, with an LD_50_ resistance ratio of 220, was collected from China, indicating that in some strains the potency of resistance to imidacloprid was continuing to increase. Since 2010, resistance to imidacloprid has continued to persist in field‐collected strains (Table 1), and is clearly entrenched in BPH populations.

**Table 2:** Dose–response data for Nilaparvata lugens laboratory susceptible (Bayer‐S) and imidacloprid‐resistant strains against imidacloprid topically applied to adult females

Strain	Year	Country	Imidacloprid	
			LD_50_ (95% limits) (ng AI insect^−1^)	RR^a^
Bayer‐S			0.61 (0.46–0.79)	1.0
CHIN‐1	2005	China	6.06 (4.82–7.55)	10.0
IND‐3	2005	India	4.47 (3.41–5.71)	7.4
IND‐5	2005	India	7.20 (6.60–7.83)	11.9
IND‐6	2005	India	11.09 (9.62–12.78)	18.3
IND‐7	2005	India	13.65 (11.42–16.07)	22.5
MAL‐1	2005	Malaysia	3.46 (3.04–3.92)	5.7
IND‐11	2006	India	58.68 (31.83–97.77)	96.7
NL2	2008	India	0.80 (0.15–2.35)	1.3
NL3	2008	India	0.86 (0.06–3.53)	1.4
NL6	2008	India	24.10 (1.15–259.09)	39.7
NL8	2008	Vietnam	2.52 (0.17–9.77)	4.2
NL9	2009	Thailand	97.00 (3.40–434.00)	139.0
NL11	2009	India	10.98 (2.18–31.00)	18.0
NL15	2009	China	20.12 (1.14–243.60)	33.1
NL16	2009	China	29.80 (5.98–64.50)	49.1
NL25	2010	India	38.88 (1.06–323.60)	64.1
NL27	2010	China	42.41 (15.61–87.62)	69.1
NL30	2010	China	133.80 (59.9–277.00)	220.4
NL32	2010	China	60.59 (31.25–85.59)	99.8

a RR = resistance ratio (R/S).

**Figure 1:**
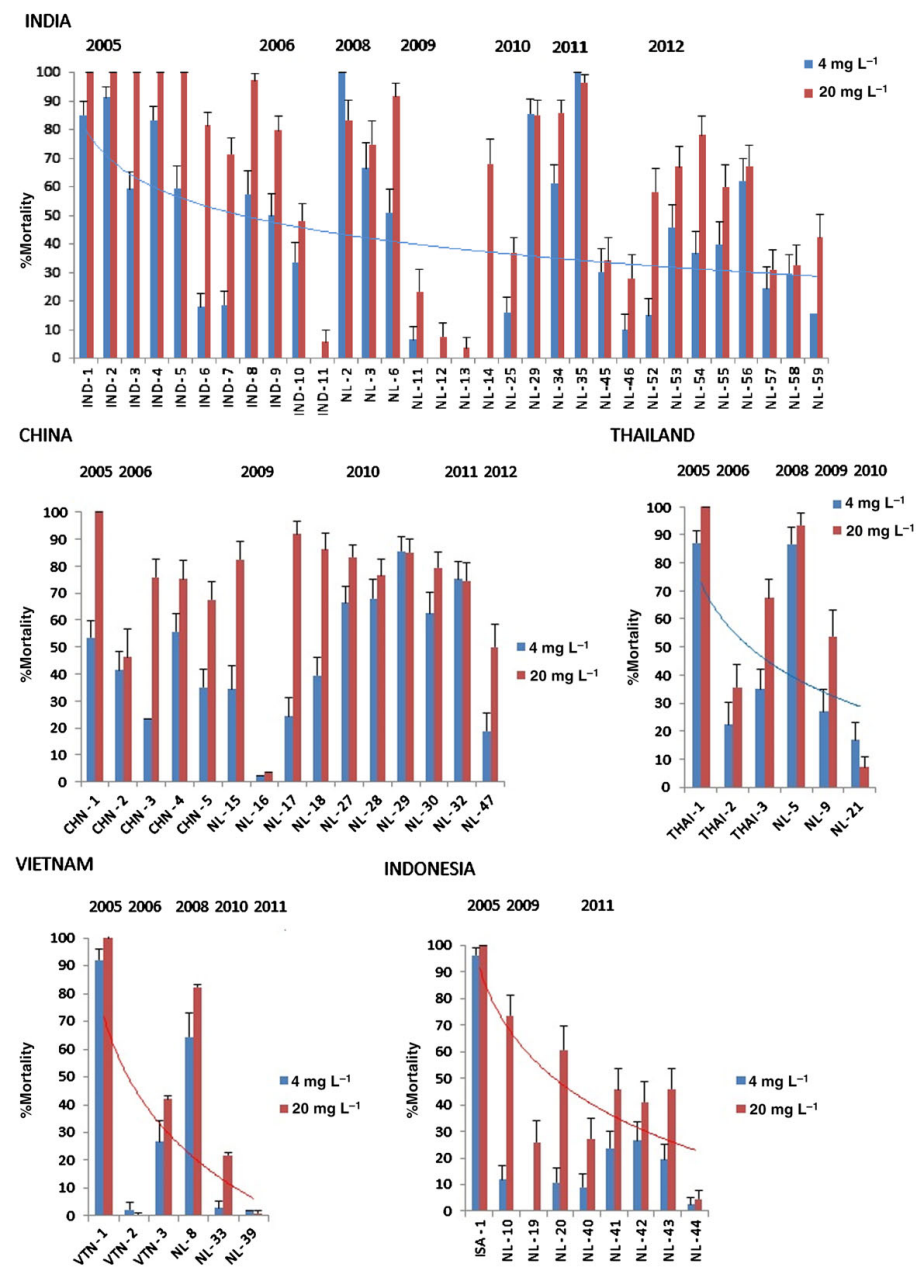
Mortalities (%) (± standard error) at two discriminating doses of imidacloprid for field‐collected strains of N. lugens.

Analysis of imidacloprid resistance development in the individual countries of India, Thailand, Indonesia and Vietnam, based on the responses of collected field strains to discriminating doses of imidacloprid, indicates a clear trend towards high resistance (Fig. 1). For China, however, the trend is less clear. This may be because BPH cannot overwinter in subtropical and temperate regions north of 22° N, and immigrate into China from other regions during the autumn months.

### 3.2 Association of overexpression of CYP6ER1 and CYP6AY1 with resistance to imidacloprid

**Figure 2:**
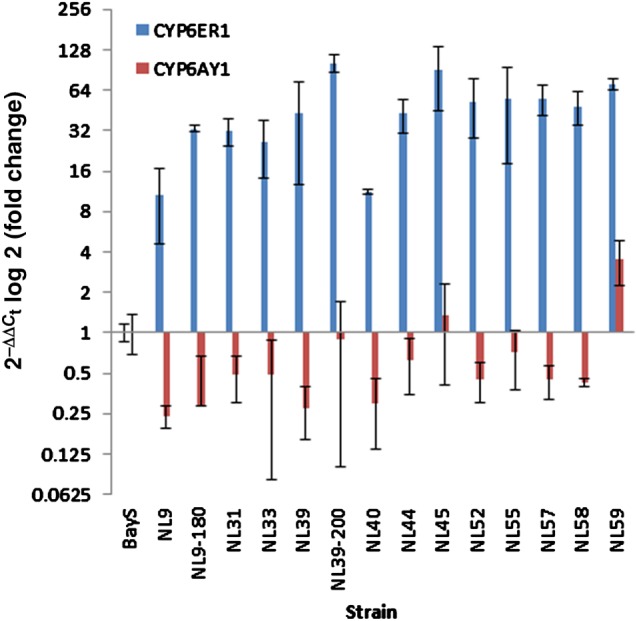
Fold change in expression of CYP6ER1 and CYP6AY1 in 14 resistant N. lugens strains compared with the susceptible reference Bayer‐S as determined by quantitative real‐time PCR. Error bars display 95% confidence intervals.

As detailed in the introduction, two cytochrome P450s have previously been linked with imidacloprid resistance in a small number of BPH laboratory and field populations. In the present study the expression levels of these two P450s were explored in 12 field populations collected from a range of countries in Asia (from 2009 to 2012) that exhibited clear resistance to imidacloprid in discriminating dose bioassays (Fig. 1, Table 1). As shown in Fig. 2, CYP6ER1 was significantly overexpressed in all 12 resistant populations when compared with a lab susceptible strain, with fold changes ranging from ten‐ to 90‐fold. In contrast, CYP6AY1 was underexpressed in ten of the populations compared with the same susceptible strain, and was only significantly overexpressed (3.5‐fold) in a single population from India (NL59). To see whether selection of the field strains with imidacloprid caused any increase in the expression levels of CYP6ER1 or CYP6AY1, two field strains (NL9 and NL39) were selected with imidacloprid up to final concentrations of 180 and 200 mg L^−1^ imidacloprid respectively. When the expression levels of CYP6ER1 were compared between NL9 (unselected) and NL9‐180 (selected), the expression level was found to have significantly increased threefold after selection, rising from ∼11‐ to 33‐fold. A similar effect was seen for NL39 (unselected) versus NL39‐200 (selected), with an increase from 43‐ to 103‐fold overexpression. Also noteworthy is that variation in the level of expression of CYP6ER1 among individual biological replicates decreased considerably after selection (as indicated by significantly reduced 95% confidence limits – see Fig. 2), suggesting that selection has reduced genetic heterogeneity in this strain and that all replicates overexpress this CYP at a universally high level. After selection, CYP6AY1 expression increased (see Fig. 2) from 0.24 in NL9 to 0.29 in NL9‐180 and from 0.28 in NL39 to 0.91 in NL39‐200; however, the difference in expression between both unselected/selected strains was not statistically significant as a result of significant variation in the expression levels of this CYP observed between biological replicates, particularly in the case of NL39‐200, and expression levels remained below that of the susceptible strain.

These results provide further evidence that overexpression of CYP6ER1 contributes to imidacloprid resistance in BPH throughout South and East Asia. The results for CYP6AY1 were surprising, and so to confirm this finding we ordered the primer pair used previously to measure CYP6AY1 expression in the study by Ding *et al.* 19 and repeated the qPCR experiments on the NL9, NL9‐180, NL39, NL39‐200 and NL59 strains. The results of this experiment confirmed our initial findings, with CYP6AY1 downregulated in all strains compared with the susceptible strain, including NL9‐180 and NL39‐200, the two selected strains (see Table 3). The previous study reporting this P450 as overexpressed used a resistant strain, originally collected from a field population in China that had been continuously selected in the laboratory with imidacloprid over 40 generations. Expression of CYP6AY1 in this strain was compared with a lab susceptible strain, and no comparison was made with the ‘unselected’ parental line of the resistant strain. However, screening of four field populations from China also showed that CYP6AY1 was significantly overexpressed (4–9‐fold). It is possible that CYP6AY1 is overexpressed in *N. lugens* populations in China and not the rest of Asia. In our study, all resistant field strains were compared with a single reference lab susceptible strain, as it is now very difficult to obtain BPH field strains that are susceptible to imidacloprid. Further investigation of the relative roles of CYP6ER1 and CYP6AY1 in imidacloprid resistance by comparing resistant strains with additional susceptible laboratory strains, or field strains if they can be sourced, is required to confirm our findings. Finally, although the results of the present study provide further evidence of a role for CYP6ER1 in imidacloprid resistance, functional characterisation of this P450 to confirm its ability to detoxify imidacloprid is now required.

**Table 3:** Fold change in expression of CYP6AY1 in five imidacloprid‐resistant N. lugens strains compared with the susceptible reference Bayer‐S as determined by quantitative real‐time PCR

Strain	Fold change ($2^{-\Delta \Delta C_{t}}$)	95% confidence level
Bayer‐S	1.06	0.45
NL9	0.30	0.10
NL9‐180	0.20	0.28
NL39	0.25	0.06
NL39‐200	0.50	0.34
NL59	0.78	0.35

### 3.3 Development of ethiprole resistance in N. lugens populations from 2005 to 2012

There was no significant variation in the responses of field samples collected in 2005 to the diagnostic concentrations of ethiprole (Table 4). Mortality of all strains was over 85% at 3 mg L^−1^ (LC_95_ of the susceptible strain) and 100% at 15 mg L^−1^ (5 × LC_95_ of the susceptible strain). In 2006, a field sample from India (IND‐11) displaying high levels of resistance to imidacloprid also survived a 3 mg L^−1^ discriminating dose bioassay with ethiprole (34% mortality), indicating an emerging resistance problem. This was confirmed in 2008, when field samples NL5 and NL8 from Thailand and Vietnam had a significant number of survivors (0 and 27% mortality respectively) when bioassayed with 3 mg L^−1^ of ethiprole. A full dose–response analysis of these two strains indicated LC_50_‐based resistance ratios for ethiprole of 67‐ and 100‐fold respectively, and 28.5‐ and 21‐fold respectively for fipronil (Table 5). In 2009, all four field samples collected from China had significant ethiprole resistance (0–14% mortality at a discriminating dose of 3 mg L^−1^), with LC_50_ resistance ratios for ethiprole ranging from 81‐ to 223‐fold and the corresponding resistance ratios for fipronil ranging from 14‐ to 68‐fold (Table 4).

**Table 4:** Mortalities (%) (± standard error) for all Nilaparvata lugens strains at two diagnostic doses (LC_95_ and 5 × LC_95_ of the susceptible strain) of ethiprole by leaf‐dip bioassay

Strain	Year	Country of origin	Region/area	Ethiprole^a^	
				3 mg L^−1^	15 mg L^−1^
Bayer‐S	—	—		100.00 ± nc	100.00 ± nc
CHN‐1	2005	China	Nanjing	100.00 ± nc	nt
IND‐1	2005	India	East Godavari District, Andhra Pradesh	98.04(±1.90)	100.00 ± nc
IND‐2	2005	India	Karnataka State	100.00 ± nc	100.00 ± nc
IND‐3	2005	India	Mumbai	96.55(±2.37)	100.00 ± nc
IND‐4	2005	India	West Godavari District, Andhra Pradesh	96.23(±2.59)	100.00 ± nc
IND‐5	2005	India	Bellary District, Karnataka State	100.00 ± nc	nt
IND‐6	2005	India	West Godavari District, Andhra Pradesh	94.90(±4.58)	100.00 ± nc
IND‐7	2005	India	East Godavari District, Andhra Pradesh	85.08(±6.23)	100.00 ± nc
ISA‐1	2005	Indonesia		98.14(±1.82)	100.00 ± nc
MAL‐1	2005	Malaysia		95.00(±4.95)	nt
THAI‐1	2005	Thailand		89.98(±4.13)	100.00 ± nc
VTN‐1	2005	Vietnam		100.00 ± nc	100.00 ± nc
IND‐11	October 2006	India	East Godavari, Andhra Pradesh	33.59(±8.35)	nt
NL2	October 2008	India	Bellary District, Karnataka State	92.00(±4.95)	nt
NL3	October 2008	India	Karnataka State	80.00(±7.30)	nt
NL5	October 2008	Thailand	Samchuk District, Suphanburi Province	0.00 ± nc	nt
NL6	October 2008	India	West Medinapuri, West Bengal, East India	83.33(±6.80)	nt
NL8	December 2006	Vietnam	Tantru District, Long An Province	26.67(±8.07)	nt
NL9	August 2009	Thailand		41.67(±9.00)	nt
NL10	September 2009	Indonesia	Subang, West Java	24.35(±7.84)	nt
NL11	October 2009	India	Sindhanoor, Southern India	77.26(±6.39)	nt
NL12	October 2009	India	Karnataka State	42.86(±8.89)	nt
NL13	October 2009	India	Nadia District, West Bengal, East India	96.72(±2.97)	nt
NL14	October 2009	India	Hooghly District, West Bengal, East India	76.13(±8.20)	nt
NL15	September 2009	China	Nanning City, Guangxi Province	0.00 ± nc	nt
NL16	September 2009	China	Danyang City, Jiangsu Province	7.41(±4.78)	nt
NL17	November 2009	China	Wuhan City, Hubei Province	14.81(±6.49)	nt
NL18	November 2009	China	Fengxin County, Jiangxi Province	7.41(±4.78)	nt
NL19	December 2009	Indonesia	East Java	23.08(±7.69)	nt
NL20	December 2009	Indonesia	Gabus Pati District, Central Java	48.53(±9.28)	nt
NL21	March 2010	Thailand	Suphanburi Province, Sriprachan District	24.29(±7.07)	nt
NL25	October 2010	India	Koppal District, Karnataka State	49.87(±7.07)	nt
NL27	September 2010	China	Danyang City, Jiangsu Province	39.20(±7.28)	nt
NL28	September 2010	China	Nanning City, Guangxi Province	42.95(±7.38)	nt
NL29	October 2010	India	West Bengal	100.00 ± nc	nt
NL30	September 2010	China	Nanchang City, Jiangxi Province	36.30(±7.17)	nt
NL32	October 2010	China	Foshan City, Guandong Province	33.58(±6.96)	nt
NL33	November 2010	Vietnam	Trà Vinh Province, Southern Vietnam	6.72(±3.82)	nt
NL34	April 2011	India	Koppal District, Karnataka State	58.35(±6.65)	nt
NL35	April 2011	India	Koppal District, Karnataka State	90.71(±4.38)	nt
NL39	August 2011	Vietnam	Hau Giang	3.64(±3.12)	0.00 ± nc
NL40	August 2011	Indonesia	Anjatan District, Indramayu	8.59(±4.55)	5.82(±3.80)
NL41	August 2011	Indonesia	Binong District, Subang	34.80(±7.94)	39.82(±7.84)
NL42	August 2011	Indonesia	Gegesik District, Cirebon	25.93(±7.30)	24.32(±7.05)
NL43	August 2011	Indonesia	Binong District, Subang	11.42(±5.30)	38.44(±8.00)
NL44	August 2011	Indonesia	Parnanukan District, Subang	34.15(±7.32)	46.12(±7.69)
NL45	September 2011	India	Raipur, Chhattisgarth	68.66(±7.43)	89.14(±4.64)
NL46	October 2011	India	Mohanpur, West Bengal	56.87(±7.47)	82.64(±5.65)
NL47	September 2011	China	Xi Jiao District, Danyang City, Jiangsu City	15.15(±5.41)	21.92(±6.17)
NL52	March 2012	India	Koppal District, Karnataka State	12.50(±5.51)	50.00(±8.33)
NL53	March 2012	India	West Godavari District, Andhra Pradesh	74.13(±7.01)	89.06(±4.88)
NL54	March 2012	India	Karimnagar, Warangal District	75.85(±6.53)	81.22(±5.96)
NL55	April 2012	India	East Godavari District, Andhra Pradesh	13.89(±5.69)	8.64(±4.68)
NL56	April 2012	India	East Godavari District, Andhra Pradesh	36.11(±7.69)	30.79(±7.69)
NL57	October 2012	India	Kanagala Camp, Karnataka State	30.00(±7.07)	65.00(±7.36)
NL58	October 2012	India	Mudhapur, Karnataka State	45.95(±8.19)	55.26(±8.07)
NL59	October 2012	India	Sidhikerra, Karnataka State	35.82(±7.78)	63.33(±7.82)

a RR = resistance ratio (R/S).

**Table 5:** Dose–response data for Nilaparvata lugens laboratory susceptible (S) and fiprol‐resistant strains against ethiprole applied as a leaf dip to adult females

Strain	Year	Country	Ethiprole		Fipronil	
			LC_50_ (95% limits)	RR^a^	LC_50_ (95% limits)	RR
Bayer‐S			0.41 (0.29–0.54)	1	1.16 (0.70–1.66)	1
NL3	2008	India	0.74 (0.52–1.06)	1.8	1.61 (1.27–2.03)	1.4
NL5	2008	Thailand	27.35 (10.56–55.50)	66.8	33.12 (9.70–76.46)	28.5
NL6	2008	India	0.21 (0.12–0.35)	0.51	1.48 (0.15–6.30)	1.3
NL8	2008	Vietnam	41.01 (13.05–116.75)	100	24.33 (6.28–79.06)	20.9
NL9	2009	Thailand	25.56 (5.23–62.57)	62.8	14.49 (7.34–27.56)	12.5
NL10	2009	Indonesia	8.06 (3.38–17.73)	19.8	50.17 (16.52–125.30)	43.3
NL11	2009	India	0.30 (0.002–1.85)	0.72	4.28 (1.79–7.79)	3.7
NL12	2009	India	21.01 (7.67–49.19)	51.6	1.45 (0.87–2.18)	1.3
NL13	2009	India	0.06 (0.00–0.26)	0.15	0.25 (0.16–0.35)	0.2
NL14	2009	India	1.06 (0.30–3.09)	2.6	2.61 (0.73–4.77)	2.3
NL15	2009	China	56.30 (29.10–108.20)	138.3	70.07 (2.35–356.30)	60.5
NL16	2009	China	90.73 (20.55–205.50)	222.9	78.41 (18.71–203.60)	67.7
NL17	2009	China	74.23 (33.43–132.80)	182.4	16.37 (14.20–18.34)	14.1
NL18	2009	China	33.06 (5.974–222.77)	81.2	16.61 (12.94–19.43)	14.3
NL19	2009	Indonesia	33.66 (3.62–105.50)	82.70	6.92 (1.27–21.85)	6.0
NL20	2009	Indonesia	42.10 (2.59–142.10)	103.4	47.71 (11.93–122.40)	41.2
NL21	2009	Thailand	13.02 (5.76–21.95)	32.0	8.21 (1.61–22.94)	7.1

a RR = resistance ratio (R/S).

For the 2010 and 2011 seasons, some apparently susceptible populations (NL29, NL35) were collected from India, but the general trend across South and East Asia indicated a developing resistance problem. Sample NL39, collected in 2011 from Vietnam (and having high levels of imidacloprid resistance), had 0% mortality at a higher (15 mg L^−1^) discriminating dose of ethiprole. Similarly, sample NL40, collected from Indonesia, also displayed good survivability (6% mortality) at the higher discriminating dose.

**Figure 3:**
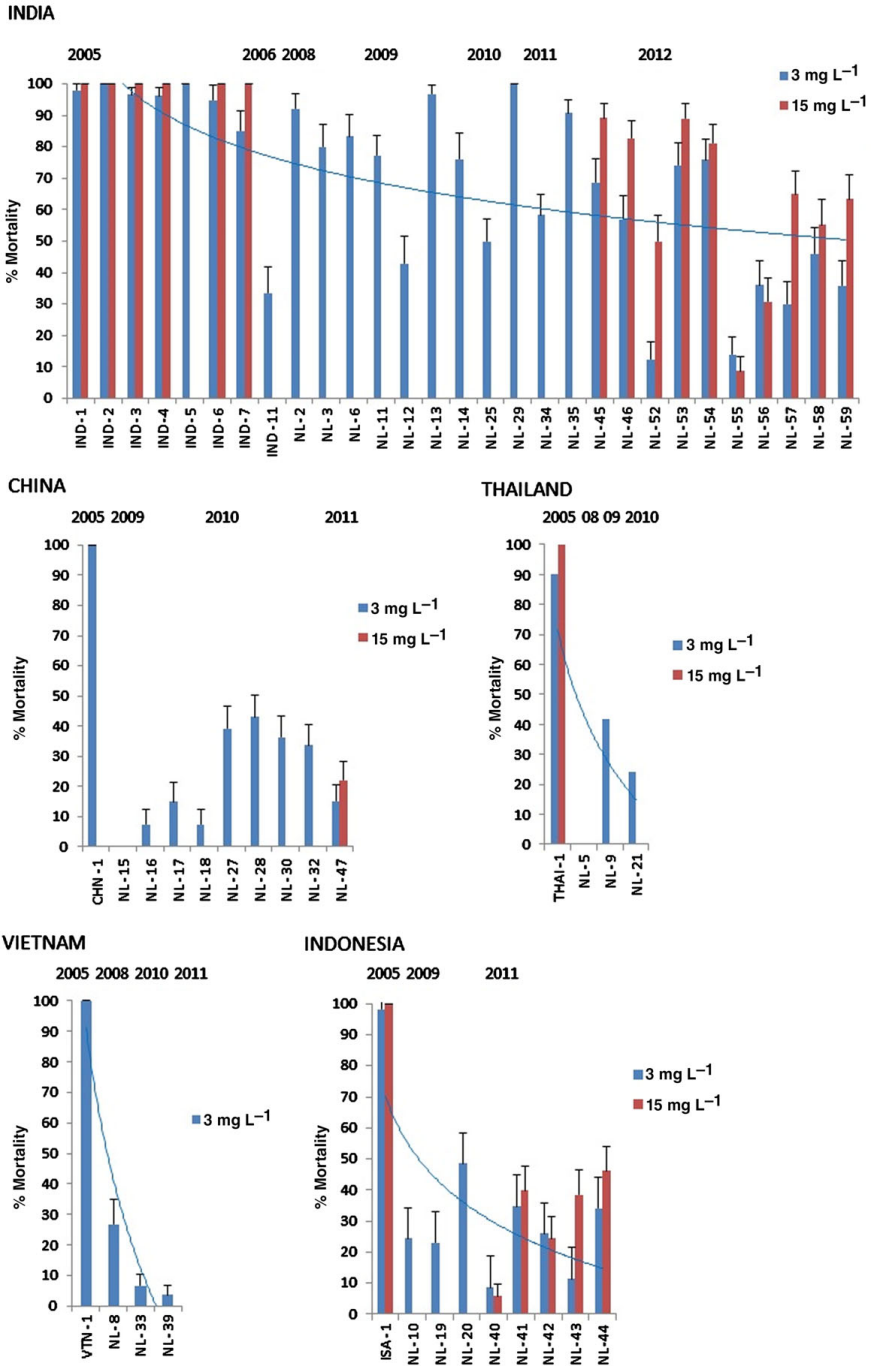
Mortalities (%) (± standard error) at two discriminating doses of ethiprole for field‐collected strains of N. lugens.

As for imidacloprid resistance, analysis of ethiprole resistance development in the individual countries of India, Thailand, Indonesia and Vietnam, based on the responses of collected field strains to discriminating doses of ethiprole (Fig. 3), indicates a clear trend towards high resistance. For China, however, the trend is again less clear, but ethiprole resistance is undoubtedly a major problem in this country.

The molecular mechanisms underlying resistance to ethiprole have not been characterised; however, work on a resistant strain from Thailand suggested that enhanced expression of P450s and esterases may contribute to resistance.^5^ Although many of the samples analysed in the present study were highly resistant to imidacloprid, there is no evidence to date for a cross‐resistance problem involving CYP6ER1.

## 4 CONCLUSIONS

At present there is no evidence of a common cross‐resistance resistance between these two chemical classes of insecticide; however, there is evidence that individual planthoppers may exhibit multiple mechanisms of resistance to the different insecticide modes of action. Our results reveal that overexpression of the cytochrome P450 CYP6ER1 is associated with imidacloprid resistance in BPH populations in five countries in South and East Asia.
